# The Preeminent Value of the Apparent Diffusion Coefficient in Assessing High-Risk Factors and Prognosis for Stage I Endometrial Carcinoma Patients

**DOI:** 10.3389/fonc.2022.820904

**Published:** 2022-02-16

**Authors:** Quan Quan, Hui Peng, Sainan Gong, Jiali Liu, Yunfeng Lu, Rongsheng Chen, Xiaoling Mu

**Affiliations:** ^1^ Department of Gynecology, the First Affiliated Hospital of Chongqing Medical University, Chongqing, China; ^2^ The Department of Obstetrics and Gynecology, Chongqing Wansheng Jingkai District Maternal and Child Health Hospital, Chongqing, China; ^3^ Department of Radiology, the First Affiliated Hospital of Chongqing Medical University, Chongqing, China

**Keywords:** endometrial carcinoma, magnetic resonance imaging, diffusion-weighted imaging, apparent diffusion coefficient, individualized management

## Abstract

**Objectives:**

To evaluate the role of the apparent diffusion coefficient (ADC) value in the individualized management of stage I endometrial carcinoma (EC).

**Methods:**

A retrospective analysis was performed on 180 patients with stage I EC who underwent 1.5-T magnetic resonance imaging. The mean ADC (mADC), minimum ADC (minADC), and maximum ADC (maxADC) values of each group were measured and compared. We analyzed the relationship between ADC values and stage I EC prognosis by Kaplan-Meier method and Cox proportional hazards analysis.

**Results:**

Patients with lower ADC values were more likely to be characterized by higher grades, specific histological subtypes and deeper myometrial invasion. The mADC, minADC and maxADC values (×10^-3^ mm^2^/s) were 1.045, 0.809 and 1.339, respectively, in grade 1/2 endometrioid carcinoma with superficial myometrial invasion, which significantly differed from those in grade 3 or nonendometrioid carcinoma or with deep myometrial invasion (0.929, 0.714 and 1.215) (P=<0.001, <0.001 and <0.001). ADC values could be used to predict these clinicopathological factors. Furthermore, the group with higher ADC values showed better disease-free survival and overall survival.

**Conclusions:**

The present study indicated that ADC values were associated with the high-risk factors for stage I EC and to assess whether fertility-sparing, ovarian preservation or omission of lymphadenectomy represent viable treatment options. Moreover, this information may be applied to predict prognosis. Thus, ADC values could contribute to managing individualized therapeutic schedules to improve quality of life.

## Introduction

Endometrial carcinoma (EC) is one of the most common malignant tumors of the female genital tract. The overall prognosis of EC is relatively good, but the incidence is increasing. In addition, patients tend to be diagnosed at a younger age (1, 2). EC has been traditionally classified into two categories, types 1: grade 1 and 2 (G1/2) endometrioid carcinoma. Types 2: grade 3 (G3) endometrioid carcinoma and nonendometrioid carcinoma (such as serous carcinoma, clear cell carcinoma and carcinosarcoma) (3). The prognosis is related to the stage and histopathologic subtype (3–5), and the 5-year survival rate is greater than 90% for stage I disease (6, 7). Surgery is the most important treatment therapy for stage I EC, and total hysterectomy and bilateral salpingo-oophorectomy and surgical staging are recommended. Follow-up supplementary treatments are determined based on the postoperative pathological results. For young patients, the quality of life after surgery is poor, especially for women of childbearing age, who desire fertility-sparing options. Some studies have suggested that conservative treatment, such as continuous progestin-based therapy, is an alternative for patients with G1 endometrioid carcinoma confined to the endometrium (8–12). In recent years, some studies demonstrated that ovarian preservation was an option for some premenopausal patients, such as patients with stage I endometrioid carcinoma, which was not associated with increased cancer-related mortality and could also avoid the risk for long-term sequelae of estrogen deprivation (13–16). In addition, an increasing number of studies have indicated that lymphadenectomy does not improve the outcome of EC patients; instead, it increases perioperative morbidities and complications, such as lymphedema, lymph cysts, pelvic nerve injury and deep venous thrombosis, resulting in a decrease in quality of life (6, 17, 18). According to the NCCN Guidelines Version 1.2020, low-risk EC is less likely to exhibit lymph node metastasis, less than 50% myometrial invasion, well or moderately differentiated histology and tumors less than 2 cm, and lymphadenectomy is not necessary. Therefore, it is important to accurately assess the high-risk factors (such as nonendometrioid, G3, and deep myometrial invasion) of patients with stage I EC before treatment, and then formulate an individualized therapeutic schedule.

However, EC is classified based on postoperative pathological results. Preoperative evaluation depends exclusively on hysteroscopy, and diagnostic curettage is not accurate. It is difficult to assess the depth of myometrial invasion and local or distant metastasis. Magnetic resonance imaging (MRI) has obvious advantages in these aspects and can evaluate the above factors relatively accurately (19, 20). In recent years, diffusion-weighted imaging (DWI) has been widely used in the evaluation of various tumors and is the only noninvasive sequence that can detect water molecule diffusion motion (Brownian motion) *in vivo*. Although DWI does not precisely distinguish edema, abscess, hematoma, benign and malignant tumors, the water molecule diffusion motion could be quantitatively measured based on the apparent diffusion coefficient (ADC). The ADC value is used to diagnose and evaluate prognosis in bladder cancer, breast cancer and prostate cancer (21–23). In previous studies, the ADC value was used to distinguish benign and malignant endometrial lesions and to evaluate the high-risk factors associated with EC (24–26). However, there are relatively few studies on the evaluation of stage I EC.

The purpose of the present study was to evaluate the high-risk factors for stage I EC based on the ADC value and explore the relationship between ADC value and disease-free survival (DFS) and overall survival (OS) for patients with EC. Then, an individualized therapeutic schedule should be managed to improve the quality of life.

## Material and Methods

This retrospective observational was approved by the Ethics Committee of our institution, and the written informed consent was obtained from all patients.

### Patients

We reviewed the clinical information obtained through the medical records. Patients with pathologically confirmed stage I EC who underwent MRI examination before surgery between August 2012 and March 2019 were retrospectively analyzed. Patients whose medical records were incomplete, such as postoperative pathological results, were excluded. Finally, a total of 180 patients (mean age = 52 ± 9 years; age range = 25–75 years) were enrolled in the study. The stage of the patients was assessed according to the International Federation of Gynecology and Obstetrics (27) staging system. Patients were followed up from the primary diagnosis to censored on September 2020 or death (median follow-up time = 33 months, range = 19-97 months). The clinicopathologic characteristics of patients are showed in **Table 1**. We divided the different groups according to the histology subtypes (endometrioid carcinoma, nonendometrioid carcinoma), depth of myometrial invasion (<½, ≥½), and tumor grade (G1, G2, G3). Group A is G1 endometrioid carcinoma confined to the endometrium. Patients of Group A could choose fertility-sparing. Group B is G2/3 endometrioid carcinoma or nonendometrioid carcinoma or with myometrial invasion. Group C is endometrioid carcinoma. Group C’s patients could choose ovarian preservation. Group D is nonendometrioid carcinoma. Group E is Stage IA G1/2 endometrioid carcinoma. Patients of Group E could choose omission of lymphadenectomy. Group F is stage IB or G3 endometrioid carcinoma or nonendometrioid carcinoma.

**Table 1:** Patients’ characteristics.

Age (mean ± SD)	(years) 52 ± 9
Variable	Data (n=180)
Postmenopausal	100 (55.56)
Type	
1	133 (73.89)
2	47 (26.11)
Histology	
Endometrioid carcinoma grade 1	48 (26.67)
Endometrioid carcinoma grade 2	85 (47.22)
Endometrioid carcinoma grade 3	18 (10.00)
Serous carcinoma	17 (9.44)
Clear cell carcinoma	11 (6.11)
Carcinosarcoma	1 (0.56)
Myometrial invasion	
Superficial	140 (77.78)
Deep	40 (22.22)

Numbers in parentheses are percentages.

### Imaging Protocol

MRI was performed using a 1.5-T unit (GE HDxt) with an eight-element pelvic phased-array surface coil. The examinations included T1-weighted imaging (T1WI), T2-weighted imaging (T2WI), and DWI. The imaging protocol included (1) axial T1WI (gradient recalled echo, GRE), T2WI (fast recovery fast spin-echo, FRFSE), DWI (spin echo echo planar imaging, SE-EPI), and enhanced T1WI (liver acquisition with volume acceleration, LAVA); (2) coronal T2WI (FRFSE) and enhanced T1WI (LAVA); and (3) sagittal T2WI (FRFSE) and enhanced T1WI (LAVA). DWI parameters were set as follows: repetition time (TR)/echo time (TE), 7500/68 ms; section thickness, 6 mm; field of views (FOV), 42 cm × 42 cm; matrix, 128 × 130; intersection gap, 2.5 mm; number of excitations, 4; and b= 0, 600 or 800 s/mm^2^.

### Imaging Analysis

**Figure 1:**
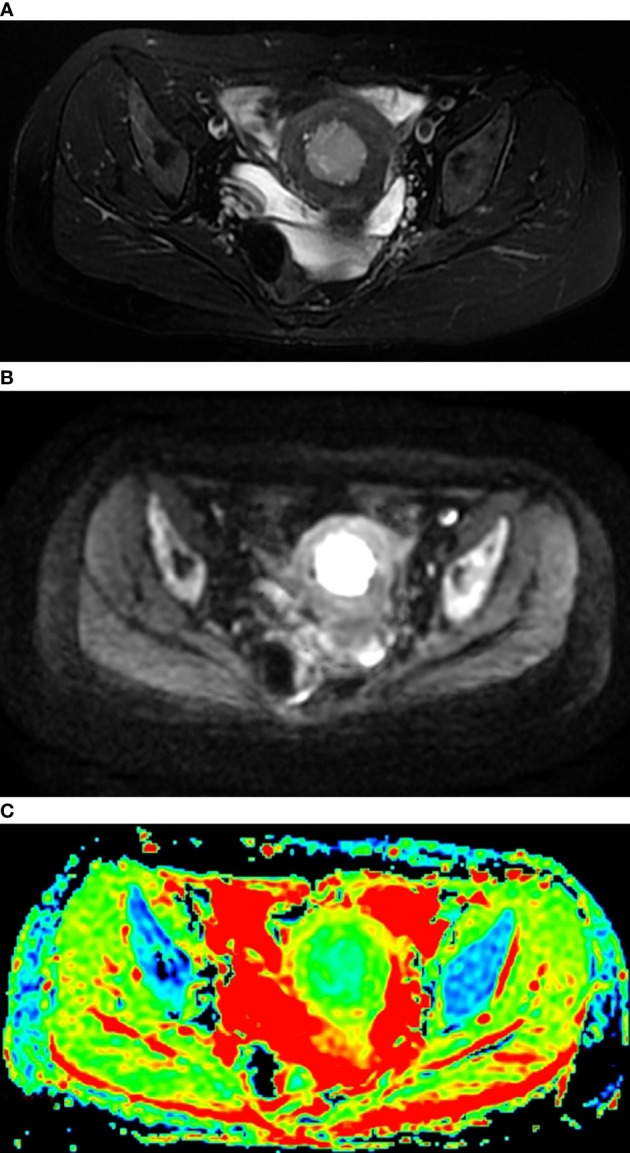
T2WI **(A)**, DWI **(B)** and DW-ADC image **(C)** of EC.

All MR sequences were evaluated by two radiologists with 10 years of experience in pelvic MRI, and the consensus was reached. The lesions were localized by T2WI and DWI with b-values of 0, 600 or 800 s/mm^2^. ADC values were measured on ADC maps based on regions of interest (ROIs) using the software (Functool) in the workstation (Advantage Window 4.6; GE) (**Figure 1**). The ROIs were carefully established in the representative solid components of tumors showing the lowest signal intensity on the ADC maps to avoid the adjacent myometrium tissue and necrosis and bleeding areas. For each lesion, a circular ROI was placed three times with sizes ranging from 40 to 100 mm^2^, and the mADC, minADC and maxADC values of the three circular ROIs were recorded. The average values of the three recordings were calculated. The mADC represented the average limitation of the diffusion of water molecules in the ROI. The minADC represented the greatest limitation of the diffusion of water molecules in the ROI, and the maxADC represented the least limitation of the diffusion of water molecules in the ROI.

### Statistical Analysis

We used SPSS software (version 25.0; IBM SPSS) for statistical analysis. The group of ADC values was described as the mean ± standard deviation (SD). The significant differences in the mean, minimum and maximum ADC values between two groups were determined using Student’s t-test or the Mann-Whitney U-test based on whether the data were normally distributed. Receiver operating characteristic (ROC) analysis was performed to assess the specificity and sensitivity of the ADC measurements. An optimal cutoff value of ADC was calculated. The Kaplan-Meier method and log-rank tests were used for analyses of disease-free survival (DFS) and overall survival (OS). Cox regression analysis was used to assess the relationship between ADC values and multivariable clinicopathological factors and DFS and OS. All the model’s assumption were tested. DFS was calculated as the number of months between the end of the primary cancer treatment and the date of recurrence, death, or the last follow-up. OS was calculated as the number of months between the date of diagnosis and that of death or the last follow-up. P< 0.05 was considered to indicate statistical significance.

## Results

### Histopathological Findings

Among the 180 patients with EC, postoperative diagnosis showed EC with superficial myometrial invasion in 140 cases and deep myometrial invasion in 40 cases. There were 151 cases of endometrioid carcinoma, 17 cases of serous carcinoma, 11 cases of clear cell carcinoma and 1 case of carcinosarcoma. The histopathological characteristics are shown in **Table 1**. During the follow-up, 8 patients were lost to follow-up. Ten patients had recurrent disease, and 5 patients died of the disease.

### Comparison of mADC, minADC, and maxADC Values Among Different Groups

**Figure 2:**
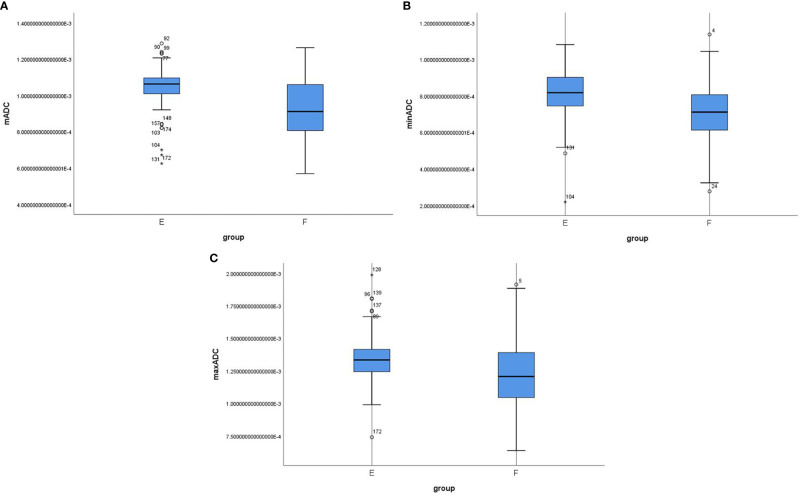
Box-whisker plot showing the correlation among mADC **(A)**, minADC **(B)** and maxADC **(C)** values in Groups E and F Group E included stage IA G1/2 endometrioid carcinoma, and stage IB or G3 endometrioid carcinoma or nonendometrioid carcinoma were included in Group F.

Among the 180 patients with EC, the mADC and minADC values were 1.082 and 0.848 (×10^-3^ mm^2^/s), respectively, for Group A (n=18) and 0.989 and 0.762, respectively, for Group B (n=162). Significant differences were noted between Groups A and B (P=0.011 and 0.024). However, the maxADC value was not significantly different (1.377 vs. 1.279, P=0.065). The mADC and maxADC values were significantly lower for Group D (n=29, 0.915 and 1.160) compared with Group C (n=151, 1.014 and 1.313) (P= 0.001 and 0.001). However, the minADC value did not differ (0.722 vs. 0.780, P=0.060). The mADC, minADC and maxADC values were significantly greater for Group E (n=107, 1.045, 0.809 and 1.339) compared with Group F (n=73, 0.929, 0.714 and 1.215) (P=<0.001, <0.001 and <0.001) (**Table 2** and **Figure 2**).

**Table 2:** The mADC, minADC, and maxADC values of the EC.

Group	n	mADC (10^-3^mm^2^/s)	minADC (10^-3^mm^2^/s)	maxADC (10^-3^mm^2^/s)
A	18	1.082 ± 0.079^*^	0.848 ± 0.110^*^	1.377 ± 0.189^*^
B	162	0.989 ± 0.146^**^	0.762 ± 0.155^**^	1.279 ± 0.224^*^
C	151	1.014 ± 0.134^*^	0.780 ± 0.151^*^	1.313 ± 0.215^*^
D	29	0.915 ± 0.164^**^	0.722 ± 0.157^*^	1.160 ± 0.220^**^
E	107	1.045 ± 0.106^*^	0.809 ± 0.134^*^	1.339 ± 0.185^*^
F	73	0.929 ± 0.162^**^	0.714 ± 0.162^**^	1.215 ± 0.252^**^

Different superscript symbols (*,**) indicate significant Differences between two groups (p < 0.05), either superscript symbols are same mean the difference is not significant (p > 0.05).

### Diagnostic Value of mADC, minADC, and maxADC in Different Groups

**Figure 3:**
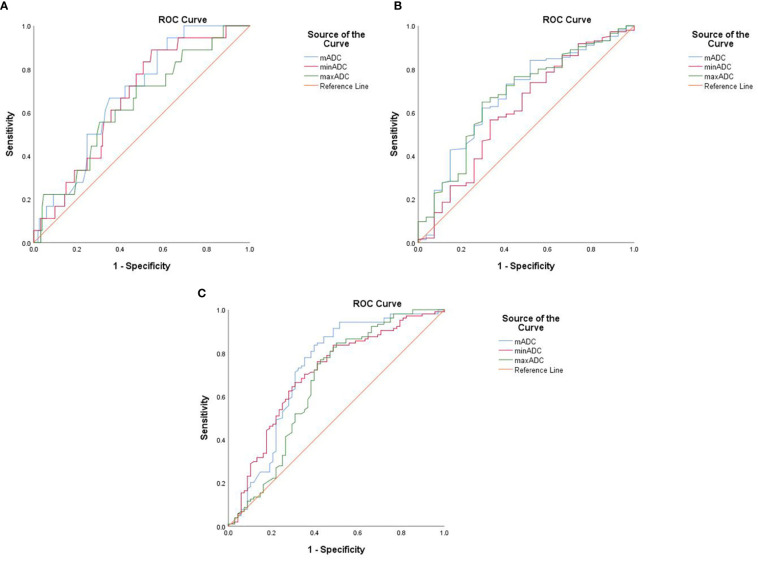
The ROC curves of mADC, minADC and maxADC values of different groups: **(A)** Groups A and B, **(B)** Groups C and D, and **(C)** Groups E and F We defined G1 endometrioid carcinoma confined to the endometrium as Group A, and the others were defined as Group B Group C included endometrioid carcinoma, whereas nonendometrioid carcinoma was defined as Group D Stage IA G1/2 endometrioid carcinoma was included in Group E, and the remaining patients were included in Group F.

According to ROC curve analysis, the areas under the curve (AUCs) were significant for mADC, minADC and maxADC predicting Groups E or F. Furthermore, the AUCs were significant for mADC and minADC predicting Groups A or B but were not significant for maxADC. The AUCs were significant for mADC and maxADC predicting Groups C or D but were not significant for the minADC. The ROC curves depicted in **Figure 3**. The sensitivity, specificity and accuracy are shown in **Table 3**.

**Table 3:** Diagnostic performance of different groups assessed by mADC, minADC and maxADC.

Groups	parameter	cut-off value (10^-3^mm^2^/s)	Sensitivity (%)	Specificity (%)	Accuracy (%)	PPV (%)	NPV (%)	+LR	-LR	AUC (95%CI)	P Value
	mADC	0.981	94.4	38.3	44.2	15.2	98.3	1.53	0.15	0.682 (0.575,0.789)	0.012
A vs B	minADC	0.756	88.9	45.5	50.0	16.0	97.2	1.63	0.24	0.662 (0.547,0.776)	0.025
	maxADC	1.382	55.6	69.5	68.0	17.5	93.0	1.82	0.64	0.633 (0.502,0.763)	0.066
	mADC	1.019	62.1	70.4	63.4	91.8	25.7	2.10	0.54	0.677 (0.564,0.791)	0.003
C vs D	minADC	0.778	56.6	66.7	58.1	90.1	22.2	1.70	0.65	0.611 (0.487,0.735)	0.067
	maxADC	1.255	64.8	70.4	65.7	92.2	27.1	2.19	0.50	0.674 (0.562,0.786)	0.004
	mADC	0.974	83.7	60.3	74.4	76.3	70.7	2.11	0.27	0.723 (0.638,0.808)	<0.001
E vs F	minADC	0.780	66.3	69.1	67.4	76.7	57.3	2.15	0.49	0.702 (0.620,0.784)	<0.001
	maxADC	1.207	84.6	50.0	70.9	72.1	68.0	1.69	0.31	0.658 (0.568,0.748)	<0.001

PPV, positive predictive value; NPV, negative predictive value; +LR, positive likelihood ratio; -LR, negative likelihood ratio; CI, confidence interval.

### Analyses of the Overall Survival and Disease-Free Survival

**Figure 4:**
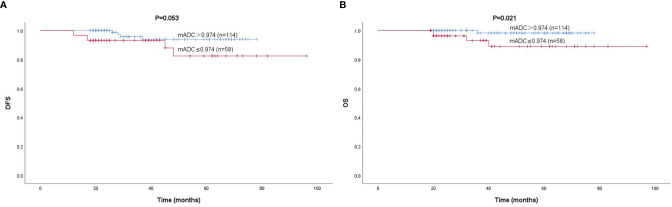
Kaplan–Meier curves for the DFS **(A)** and OS **(B)** of 172 patients, according to mADC. The cutoff value was 0.974×10^-3^ mm^2^/s.

According to the cutoff value, patients with a lower mADC were associated with worse DFS and OS, and patients with a higher mADC were associated with better DFS and OS (**Figure 4**). Unfortunately, the ADC values were not independent prognostic factors of DFS and OS (**Table 4**).

**Table 4:** Multivariate prognostic analyses.

Parameters	HR	DFS 95%CI	p	HR	OS 95%CI	*p*
Age						
≤60 y	1			1		
>60 y	0.528	0.072-3.893	0.531	0.447	0.022-9.081	0.601
Menopause						
No	1			1		
Yes	1.301	0.250-6.784	0.754	1.112	0.079-15.676	0.937
Type						
1	1			1		
2	4.827	1.017-22.904	0.048	6.621	0.574-76.322	0.130
Myometrial invasion						
Superficial	1			1		
Deep	0.853	0.149-4.883	0.858	2.509	0.258-24.426	0.428
mADC (10^-3^mm^2^/s)						
≤0.974	1			1		
>0.974	0.633	0.045-8.976	0.735	0.113	0.001-10.526	0.346
minADC (10^-3^mm^2^/s)						
≤0.780	1			1		
>0.780	0.518	0.061-4.387	0.546	1.997	0.030-133.498	0.747
maxADC (10^-3^mm^2^/s)						
≤1.207	1			1		
>1.207	1.689	0.201-14.209	0.630	1.844	0.147-23.102	0.635

HR, hazard ratio; CI, confidence interval.

## Discussion

In our study, we found that the mADC and minADC values were significantly increased in patients with G1 endometrioid carcinoma confined to the endometrium, whereas the maxADC value showed no significant difference. The mADC and maxADC values were significantly lower in patients with nonendometrioid carcinoma, whereas the minADC value did not significantly differ. The mADC, minADC and maxADC values were all significantly increased in patients with stage IA G1/2 endometrioid carcinoma. The data showed that the ADC values could be used to assess the high-risk factors for stage I EC, such as deep myometrial invasion and G3, and therefore applied to the risk stratification of EC to formulate personalized treatment for improving the quality of life of patients. Furthermore, patients with higher mADCs were prone to better DFS and OS; otherwise, patients with lower mADCs were prone to worse DFS and OS. The data showed that the ADC values were related to tumor prognosis.

DWI is a noninvasive MRI functional sequence that reflects the diffusion motion of water molecules. DWI can indirectly reflect the microscopic changes of tissues and cells by reflecting the limitation of diffusion motion of water molecules in tissues. For example, compared with normal tissue, tumors exhibit increased cell density, enlarged nuclei, increased macromolecular protein content, and decreased extracellular space, resulting in restricted water molecule diffusion motion. For different grades of tumors, the higher the grade of the tumor, the greater the cell density, which leads to the restriction of the diffusion motion of water molecules (28). However, some studies have shown that the resolution of DWI is relatively low with a high signal for tumors and some benign lesions, such as edema, abscess and hematoma. Therefore, DWI could not be used as a tool to distinguish benign and malignant lesions or tumor staging alone. However, the ADC values obtained from DWI could quantitatively assess the diffusion motion of water molecules, thereby distinguishing benign and malignant lesions and evaluating the heterogeneity of tumors more accurately. Previous studies have demonstrated that ADC can be used to identify deep or superficial myometrial invasion. In addition, some studies have shown that as the grade of the tumor increases, the ADC values decrease accordingly. Other studies are also consistent, and ADC could be used to predict tumor grade and lymph node metastasis (29–33). Previous studies have also shown that ADC could be used to distinguish between type 1 and 2 EC (34). However, no studies have evaluated these factors together to assess whether patients with stage I EC could choose fertility-sparing, ovarian preservation or omission of lymphadenectomy. Thus, patient prognosis can be improved, and the quality of life can be improved.

Most patients with EC are diagnosed at an early stage and receive standard treatment, so the prognosis is relatively good (35). However, in previous studies, approximately 14% of patients were premenopausal, and 5% of patients were younger than 40 years (36). The standard treatment of EC includes bilateral oophorectomy, which is mainly based on three theories. First, estrogen produced by the ovaries may activate residual microscopic EC. In a previous *in vitro* study, estrogen stimulated the growth of EC cells and upregulated the expression of estrogen receptors (37). However, estrogen has not been proven to have an effect on clinical data until now. In addition, there are some reports about the use of estrogen replacement therapy in postmenopausal patients with EC, and these patients have no increased risk of recurrence or death after receiving treatment. A prospective study conducted by the Gynecologic Oncology Group also showed that the absolute recurrence rate of patients with EC who received estrogen replacement therapy was only 2.1% (13). Second, there is a risk of ovarian metastasis, and synchronous ovarian primary tumors may cause recurrence. The probability of ovarian invasion of early EC is approximately 5%, and most of the ovaries are abnormal or accompanied by extrauterine lesions, which could be detected by preoperative imaging examination or intraoperatively. In addition, microscopic ovarian lesions are noted in less than 1% of patients. Third, these patients with EC may have an increased risk of primary tumors in the future due to potential mutations in important genes, such as BRCA, and gene mutations associated with Lynch syndrome. However, studies have shown that the incidence of Lynch syndrome in EC is only 5–9%. Patients with EC even had lower rates of BRCA mutations compared with the normal population (37). Nevertheless, for patients who choose ovarian preservation, we still need to pay attention to the molecular subgroups characterized at the preoperative diagnosis. Furthermore, premature removal of the ovaries could cause not only menopausal symptoms, such as hot flashes, but also an increased risk of osteoporosis, cardiovascular disease and cognitive dysfunction. Some studies showed that undergoing bilateral oophorectomy before 35 years could increase the risk of myocardial infarction by greater than 7-fold. Women who underwent bilateral oophorectomy before 55 years exhibited an 8.6% increase in mortality (13). Therefore, ovarian preservation is safe and beneficial for patients with stage I endometrioid carcinoma. Numerous studies have demonstrated that ovarian preservation does not affect survival in patients with stage I endometrioid carcinoma (15).

For women of childbearing age, hysterectomy is even more unacceptable. Some studies have suggested that conservative treatment could represent an alternative for patients with G1 endometrioid carcinoma confined to the endometrium. Progestin-based therapy was effective for most patients with G1 endometrioid carcinoma confined to the endometrium. Even if the effect was poor or the disease recurred, the tumor rarely extends beyond the uterus (38). In addition, hysteroscopic resection of the tumor has been proposed as another strategy for fertility-sparing. However, this treatment is only limited to case reports, and it is unclear whether hysteroscopic resection of the tumor could improve the prognosis (39).

Recently, an increasing number of studies have suggested that lymphadenectomy is not recommended for low-risk EC. The NCCN Guidelines Version 1.2020 also recommend lymphadenectomy for patients with high-risk EC. For low-risk EC, omission of lymphadenectomy did not worsen DFS or OS. It could also decrease perioperative morbidities and complications, such as lymphedema, gastrointestinal injury and lymphocysts. In addition, lymphadenectomy can increase the possibility of blood transfusion, increase the average duration of surgery, and cause a longer hospital stay, thus affecting the quality of life of patients (6).

Therefore, an accurate assessment of the risk stratification of patients with stage I EC before treatment is crucial for the formulation of an individualized therapeutic schedule. Previous studies have shown that the ADC values could be used to distinguish G3, deep myometrial invasion, lymph node metastasis and other high-risk factors (29, 31, 33, 40). In our study, the ADC values were significantly increased in groups treated with an alternative method for uterine or ovarian preservation or omission of lymphadenectomy compared with standard of care groups. The ADC cutoff values obtained from ROC curves could be used to differentiate the various groups. Thus, pretreatment ADC values are used to provide more accurate evaluations for patients with stage I EC and then manage personalized treatment, improving the quality of life of patients. We also found that lower ADC values were associated with worse DFS and OS. Unfortunately, the ADC values were not an independent prognostic factor of DFS and OS, but there is currently no superior preoperative biomarker to replace the preeminent value of ADC to assess the high-risk factors associated with stage I EC. Therefore, pretreatment ADC values may represent a potential biomarker for predicting prognosis.

Our study had some limitations. First, this study is a retrospective analysis that lacks an acknowledged standardized measurement method for ADC. The cohort should be multicenter, and larger studies are needed in the future to develop a standardized procedure. Another limitation may lie in the fact that the distribution of ADCs is calculated by a “single-layer model” rather than the overall volume of the tumor. Volume assessment is time-consuming and therefore difficult to perform in daily clinical practice.

## Conclusion

The ADC values combined with DW-MRI could be used to evaluate and predict clinicopathological factors of stage I EC and to assess whether fertility-sparing, ovarian preservation or omission of lymphadenectomy represent viable treatment options. Moreover, higher ADC values were related to better DFS and OS. Thus, the pretreatment ADC value may represent a potential biomarker to predict the aggressiveness and prognosis of patients with EC, which contributes to managing initial personalized treatment to improve the quality of life.

## Data Availability Statement

The raw data supporting the conclusions of this article will be made available by the authors, without undue reservation.

## Ethics Statement

The studies involving human participants were reviewed and approved by the Ethics Committee of the First Affiliated Hospital of Chongqing Medical University. The patients/participants provided their written informed consent to participate in this study.

## Author Contributions

QQ and XM designed the study. QQ drafted the manuscript. HP and JL conducted the statistical analysis. YL and RC collected the data. HP and SG were responsible for follow-up. XM revised the manuscript. All authors have read and approved the final study.

## Conflict of Interest

The authors declare that the research was conducted in the absence of any commercial or financial relationships that could be construed as a potential conflict of interest.

## Publisher’s Note

All claims expressed in this article are solely those of the authors and do not necessarily represent those of their affiliated organizations, or those of the publisher, the editors and the reviewers. Any product that may be evaluated in this article, or claim that may be made by its manufacturer, is not guaranteed or endorsed by the publisher.
